# Dimethyl Fumarate Combined With Vemurafenib Enhances Anti-Melanoma Efficacy *via* Inhibiting the Hippo/YAP, NRF2-ARE, and AKT/mTOR/ERK Pathways in A375 Melanoma Cells

**DOI:** 10.3389/fonc.2022.794216

**Published:** 2022-01-24

**Authors:** Hongxia Li, Yaping Wang, Rina Su, Yuchen Jia, Xiong Lai, Huimin Su, Yaochun Fan, Yuewu Wang, Wanjin Xing, Jianzhong Qin

**Affiliations:** ^1^ Inner Mongolia Key Laboratory for Molecular Regulation of the Cell, College of Life Sciences, Inner Mongolia University, Hohhot, China; ^2^ Inner Mongolia Autonomous Region Center for Disease Control and Prevention, Hohhot, China; ^3^ College of Biological Sciences and Biotechnology, Dalian University, Dalian, China

**Keywords:** melanoma, dimethyl fumarate, vemurafenib, combination therapy, transcriptomic, reactive oxygen species, yes-associated protein

## Abstract

Melanoma is a deadly form of skin cancer with high rates of resistance to traditional chemotherapy and radiotherapy. BRAF inhibitors (BRAFi) can achieve initial efficacy when used to treat melanoma patients, but drug resistance and relapse are common, emphasizing the need for new therapeutic strategies. Herein, we reported that combination of dimethyl fumarate (DMF) and vemurafenib (Vem) inhibited melanoma cell proliferation more significantly and induced more cell death than single agent did both *in vitro* and *in vivo*. DMF/Vem treatment induced cell death through inhibiting the expression and transcriptional activity of NRF2 thereby resulting in more reactive oxygen species (ROS) and *via* inhibiting the expression of YAP, a key downstream effector of Hippo pathway. DMF/Vem treatment also reduced phosphorylation of AKT, 4EBP1, P70S6K and ERK in AKT/mTOR/ERK signaling pathways. RNA-seq analysis revealed that DMF/Vem treatment specifically suppressed 4561 genes which belong to dozens of cell signaling pathways. These results indicated that DMF/Vem treatment manifested an enhanced antitumor efficacy through inhibiting multiple cell signaling pathways, and thus would be a novel promising therapeutic approach targeted for melanoma.

## Introduction

Malignant melanoma is a highly aggressive form of skin cancer that arises when melanocytes accumulate particular oncogenic mutations (1, 2). While relatively rare, melanoma is nonetheless the cause of most skin cancer-related mortality at present (3). This is in part because these tumors are prone to rapid metastasis, and metastatic melanoma is associated with a poor prognosis and median survival duration of just 6-9 months (4). Early-stage cutaneous melanoma tumors can be cured *via* surgical excision, whereas metastatic melanoma cannot be readily cured owing to the high metastatic burden and the difficulty of detecting these metastases using imaging tools or accessing them surgically (5). Systemic treatments for melanoma patients include a combination of chemotherapy, targeted therapies (MAPK pathway inhibitors), and immunotherapies (immune checkpoint inhibitors) (6, 7). However, many of these melanoma treatments are subject to substantial limitations (8). Dacarbazine (DTIC), for example, has been approved for the chemotherapeutic treatment of melanoma, but it only yields some level of response in 15-20% of treated individuals (9). While the advents of targeted therapies and immunotherapeutic regimens have significantly prolonged melanoma patient overall survival, treatment failure is nonetheless common and both the acquisition of drug resistance and severe adverse events are common.

The MAPK signaling pathway serves as a central regulator of essential cellular processes including proliferation, differentiation, migration and apoptosis. Mutations which result in aberrant MAPK pathway activation most often drive melanoma tumorigenesis (10). Roughly half of all cutaneous melanomas carry activating mutations in the *BRAF* gene which encodes for a key MAPK signaling protein, of these mutations 90% are the *BRAF^V600E^* (11, 12). This finding lead to the development of small-molecule inhibitors of mutant BRAF, including vemurafenib (Vem), dabrafenib, and encorafenib (13, 14). Vem has been approved by the Food and Drug Administration (FDA) for the targeted treatment of unresectable or metastatic melanomas harboring activating BRAF*^V600E^* mutation (15), and tumor regression has been reported in 90% of patients treated with Vem (6, 15). However, melanomas tend to acquire Vem resistance rapidly through MAPK pathway reactivation and PI3K-AKT-mTOR pathway activation, or other mechanisms, ultimately constraining the therapeutic utility of this inhibitor (7). The common mechanisms that result in MAPK reactivation and persistent ERK signaling typically include changes in BRAF, NRAS, MEK, and neurofibromin 1 (NF1) (16, 17). Thus MEK inhibitors (MEKi) including cobimetinib, trametinib, and binimetinib have been designed to overcome the BRAF inhibitor (BRAFi)-related MAPK pathway reactivation. Combination of cobimetinib with Vem was first approved in 2015 for treating melanomas harboring BRAF mutations and were not eligible for surgical excision or were metastatic (18). Recently, combination of BRAFi with MEKi therapeutic regimens (vemurafenib/cobimetinib, dabrafenib/trametinib, and encorafenib/binimetinib) have been employed as the first-line therapeutic options for individuals diagnosed with BRAF-mutant metastatic melanoma (19–21), resulting in median progression-free survival (PFS) and overall survival (OS) durations of 11-15 and 22-33 months, respectively, for treated individuals (19, 21, 22). Among these, the combined treatment of dabrafenib with trametinib gave rise to a 5-year survival rate of 34% of individuals (23). However, these combination treatment regimens were commonly followed by acquisition of resistance over a period of several months (24), and even have no effect in 15-20% of patients harboring BRAF*^V600E^* mutations (25). It is thus critical for novel drugs or combinations to be identified in order to further improve clinical outcomes in malignant melanoma patients (26).

Dimethyl fumarate (DMF; trade name: Tecfidera) is a drug that has been registered for the treatment of relapsing-remitting multiple sclerosis and psoriasis, exhibiting satisfactory safety characteristics (http://www.fda.gov) (27). It was first reported in 2006 that DMF treatment was sufficient to induce anti-proliferative and pro-apoptotic effects which constrained melanoma growth and metastasis *in vitro* and *in vivo* (28). Recent work has further confirmed that DMF can inhibit the invasive and metastatic activity of melanoma cells by suppressing matrix metalloproteinase expression (29, 30). More recent preclinical evidences also suggested that DMF might exhibit potential antitumor activity when used to treat melanoma (28, 29), breast cancer (31), colon cancer (32, 33), ovarian cancer (34), and lung cancer (32). Moreover, a combination of DTIC and DMF significantly reduced lymph vessel density in primary tumors and impaired melanoma cell migration *in vitro* (35). DMF has been found to target a range of pathways involved in cancer pathogenesis to achieve antitumor efficacy, including nuclear factor erythroid 2 (NF-E2)-related factor 2 (NRF2), protein deglycase DJ-1/Parkinson disease protein 7 (DJ-1), and extracellular signal-regulated kinase 1 and 2 (ERK1/2) (27). Despite these findings, further researches are needed to understand the context- and cancer type-dependent mechanisms underlying the anti-tumor effects of DMF treatment. In particular, the anti-melanoma activities of DMF triggered our interest in exploring the effects and mechanisms of combined treatment of DMF with BRAF*^V600E^* inhibitors for melanoma in an effort to design novel clinical combinational regimens for improving melanoma patient outcomes.

Herein, we evaluated the anti-melanoma efficacy of DMF/Vem treatment and explored the mechanisms underlying such efficacy. We found that DMF/Vem treatment was associated with significantly enhanced suppression of melanoma cell proliferation and increased apoptotic tumor cell death *in vitro* and *in vivo*. These anticancer activities were at least partially attributable to the induction of a burst of reactive oxygen species (ROS) production and concomitant inhibition of the Hippo/YAP, NRF2-ARE, and AKT/mTOR/ERK signaling pathways.

## Materials and Methods

### Cell Culture and Reagents

Human A375 cutaneous melanoma cells were obtained from the Cell Bank of the Chinese Academy of Sciences (Shanghai, China), and were cultured in DMEM (Gibco, USA) containing 10% (v/v) fetal bovine serum (FBS; Gibco), 100 μg/mL streptomycin (Gibco), and 100 U/mL penicillin (Gibco), in a 5% CO_2_ humidified incubator (Sanyo, Japan) at 37°C.

Dimethyl fumarate (DMF) and tert-butyl hydro-peroxide (t-BHP) were purchased from Sigma Chemical Co. (Sigma-Aldrich Ltd., USA), while vemurafenib (Vem) was from Selleck Chemicals (TX, USA), 5-Chloro-methyl fluorescein diacetate (CMFDA) was from Invitrogen (CA, USA), 2′,7′-Dichlorofluorescein diacetate (DCFH-DA) and brusatol were from Beyotime Biotechnology (Shanghai, China) and Shanghai Yuanye Bio-Technology (Shanghai, China), respectively.

### CCK-8 Assay

Cell viability was assessed *via* CCK-8 assay. Briefly, A375 cells were added to 96-well plates (7×10^3^/well) and were treated with a range of DMF concentrations and/or with Vem (2 μM) or DMSO as a control for 48 h after an initial overnight culture period. Next, 10 µL of CCK-8 reagent (Dojindo, Japan) was added into each well, and plates were incubated for an additional 1 h prior to the measurement of optical density (OD) values of each well at 450 nm using a microplate reader (BioTEK, Saxony, USA). Relative cell proliferation rates were then calculated by dividing OD values from experimental cells by those from control cells.

### Colony Formation Assay

A total of 500 A375 cells were added per well of a 6-well plate and were treated for 72 h with compounds of interest following an initial overnight incubation. The media in each well was then changed every other day for a two-week period, the cells then were fixed with 4% paraformaldehyde (PFA; Sangon Biotech, Shanghai, China) and stained with crystal violet (Beyotime) for photographing. Finally colonies were dissolved with 2% SDS, and the OD value for each sample at 570 nm was determined *via* microplate reader (BioTEK).

### Flow Cytometry

To assess cell survival, A375 cells were added to 6-well plates (2×10^5^/well) and incubated overnight, then were treated with 2 μM Vem and/or 50 μM DMF for 24 h. Cells were then collected, washed two times in PBS, stained for 30 min with FITC/APC-conjugated annexin V and propidium iodide (PI), and evaluated with a flow cytometer (BD FACS AriaIII, USA).

To analyze cell cycle distributions, A375 cells (2×10^5^/well) were treated with 2 μM Vem and/or 50 μM DMF for 24 h, then were harvested, fixed in chilled 70% ethanol, incubated in 25 µg/mL RNase A at 37°C for 20 min. The cells were resuspended in 50 µg/mL propidium iodide for assessment by flow cytometry (BD FACS AriaIII), and the data were analyzed by FlowJo vX.0.7.

Cellular ROS levels were assessed with a Fluorometric Intracellular ROS Kit (Beyotime) based on provided directions. Briefly, cells were added to a 6-well plate (2×10^5^/well) and incubated overnight, after which they were treated for 24 h with Rosup (as a positive control), 2 μM Vem, and/or 50 μM DMF. Cells were then stained for 30 min with 10 µM DCFH-DA in the serum-free media at 37°C, after which they were analyzed *via* flow cytometry (BD FACS AriaIII).

### Immunofluorescent Staining

Cells were applied to slides and fixed with 4% paraformaldehyde for 15 min, after which they were rinsed in PBS, permeabilized with 0.2% Triton X-100 for 5 min, and blocked at room temperature in 2% BSA for 30 min. Slides were then incubated with anti-YAP (D8H1X, Cell Signaling Technology, MA, USA) or anti-NRF2 (ab62352, Abcam, Cambridge, UK) antibodies, rinsed with PBS, probed with appropriate Cy3-labelled secondary antibodies, and subjected to nuclear counterstaining using Hoechst 33258 (Sigma-Aldrich Ltd.). Fluorescence was then visualized with a Zeiss LSM710 confocal microscope (Zeiss, Oberkochen, Germany).

### Quantitative Real-Time Reverse Transcription PCR (qRT-PCR)

Trizol (Takara, Japan) was used to extract total RNA from prepared cells, and the PrimeScript^®^ RT reagent Kit (Takara) was used to prepare cDNA. All qRT-PCR reactions were conducted with primers compiled in the **Supplementary Table 1** and the SYBR^®^ Premix Ex Taq™ kit (Takara) in a LightCycler^®^ 480 instrument (Roche Applied Science, Germany). Relative gene expression was assessed *via* the 2^-ΔΔCt^ method, with *18S* as a normalization control. Experiments were performed in triplicate.

### Western Blotting

The M-PER^®^ Mammalian Protein Extraction Reagent (Thermo Fisher Scientific Inc., USA) supplemented with complete protease inhibitor (Roche Applied Science, Germany) and phosphatase inhibitor (Sigma-Aldrich Ltd., USA) cocktails were used to extract total protein from samples of interest, whereas a Nuclear and Cytoplasmic Protein Extraction Kit (KeyGEN, China) was used to collect proteins from these specific subcellular compartments. A BCA kit (Beyotime) was used to quantify the protein concentrations of these samples, then the proteins were separated *via* SDS-PAGE and transferred onto 0.22 μm polyvinylidene difluoride (PVDF) membranes (Millipore Ltd., USA). The membranes were blocked in 5% non-fat milk, and were then probed with appropriate primary and secondary antibodies (**Supplementary Table 2**). Finally the proteins were detected using an enhanced chemiluminescent detection kit (Thermo Fisher Scientific Inc., USA), with GAPDH or β-actin serving as loading controls.

### Dual-Luciferase Reporter Assay

A375 cells were transfected with the pGL3-promoter plasmid carrying the antioxidant response element (ARE) to assess ARE-dependent firefly luciferase expression, together with the control pRL-TK plasmid encoding TK Renilla luciferase to evaluate transfection efficiency. After 36 h of transfection, cells were treated with appropriate compounds for 12 h, with Brusatol and t-BHP serving as negative and positive controls, respectively. Lysates were then collected from these cells, and the firefly and Renilla luciferase activity therein were determined with a Dual-Luciferase Reporter Assay System (Promega). Experiments were performed in triplicate, and Renilla luciferase activity was used for the normalization of firefly luciferase activity.

### YAP Overexpression

YAP was overexpressed in A375 cells by transfecting them with the pcDNA3.1 (+)-YAP overexpression plasmid or the pcDNA3.1 (+) control vector (both from Genechem Company, Shanghai, China) using Lipofectamine 2000 (Invitrogen). At 48 h of post-transfection, cells were treated with 2 μM Vem and 50 μM DMF for 24 h, then cell death was assessed *via* Annxin V/PI staining and flow cytometry as above, while YAP protein levels were assessed *via* western blotting.

### Transcriptomic Analysis

Total RNA was extracted from A375 cells treated with DMF and/or Vem for 24 h using the Trizol reagent (Invitrogen, CA, USA) and quality of RNA samples were controlled by RNA purity (1.8 < OD260/OD280 < 2.1), RNA integrity (RNA Integrity Number, RIN > 9.0) and RNA concentration. A total amount of 3 µg RNA per sample was used as input material for the RNA sample preparations. Sequencing libraries were generated using NEBNext^®^ Ultra™ RNA Library Prep Kit for Illumina^®^ (NEB, USA) following the manufacturer’s recommendations and index codes were added to attribute sequences to each sample. Then the sequencing was performed on the Illumina NovaSeq 6000 instrument (Illumina, USA) and 150 bp paired-end reads were generated. Finally, Hisat2 was used for sequence alignment. Fragments per kilobase of transcript per million mapped reads of known genes were calculated using eXpress v1.5.1. The database construction and sequencing services were provided by Novogene (Beijing, China). Differentially expressed genes (DEGs) were identified by comparing sequencing results from different samples, and were visualized *via* a principal component analysis (PCA) approach. Genes with an adjusted P-value (*padj*) < 0.05 found by DESeq2 were assigned as differentially expressed. All multivariate statistical analyses and result visualizations were conducted using the OmicShare tools (www.omicshare.com/tools).

### Animals

Female BALB/c nude mice (6 weeks old, ~20 g) were obtained from Beijing Vital River Laboratory Animal Technology Co., Ltd (Beijing, China), and were housed in a specific pathogen-free climate-controlled facility (n=5/cage) with free food and water access. All animal experiments were approved by the Ethics Committee of the Inner Mongolia University (reference no. IMU-MO-2020-011).

### Xenograft Tumor Models

We firstly conducted a pilot study to calculate the standard deviation among animals in this study. Based on power analysis (36) at 80% power (*β* = 0.20), with 95% confidence (*α* = 0.05), we chose the sample size as 10 mice per group, then subcutaneously implanted A375 cells (2×10^6^ in 100 µL PBS) on the right lateral flank of each mouse. When tumors grew to ~200 mm^3^ in size, those mice were randomly assigned into four subgroups and treated by intratumoral injection of different drugs respectively every other day (**Figure 2A**). The four subgroups included (1) DMF group (DMF only at 6 mg/kg body weight) (2);Vem group (Vem only at 25 mg/kg body weight) (3);DMF plus Vem group (DMF at 6 mg/kg and Vem at 25 mg//kg body weight), and (4) vehicle control group (10 µL of a mixture of DMSO-PEG400-PBS). Tumor volumes were measured with calipers, and the mice body weights were recorded every other day. Tumor volume (V) was determined as follows: (D×d^2^)/2, where D and d correspond to the long and short tumor diameters, respectively. The differences between the groups were analyzed with one-way analyses of variance (ANOVAs). On day 10 after the initiation of treatment, mice were euthanized. Part of tumor tissue from each mouse was fixed with 4% PFA and embedded in paraffin for histological analysis, while the remaining tumor tissue was snap-frozen in liquid nitrogen.

### Immunohistochemistry Analysis

The tumor sections (4 μm) were deparaffinized in xylene and rehydrated in gradients of alcohol-H_2_O, antigen retrieval was then performed using a microwave. Subsequently, the endogenous peroxidase activities and the non-specific protein binding were blocked with 3% H_2_O_2_ for 25 min and normal goat serum (10%) for 30 min, respectively. The sections were then incubated with specific primary antibodies against pERK (1:200) and YAP (1:200) overnight at 4°C, followed by secondary antibody for 30 min. Then, the sections were stained and lightly counter-stained with diaminobenzidine (DAB) chromogen and hematoxylin, respectively. Immuno-histochemical positive stained cytoplasm showed brown and hematoxylin-stained nuclei showed purple. The integrated optical density (IOD) of the immunohistochemistry (IHC) section was calculated by Image Pro Plus 6.0 (37).

### Statistical Analysis

GraphPad Prism v7.0 (CA, USA) was used for all statistical testing, data being compared between groups through two-tailed Student’s t-tests and one-way analyses of variance (ANOVAs) unless stated otherwise. Data were given as mean ± standard deviation (SD) unless stated otherwise. The results are presented as the average of at least 3 independent experiments, and *P* < 0.05 was the threshold of significance (*P < 0.05, **P < 0.01, ***P < 0.001, ****P < 0.0001).

## Results

### Combination of DMF With Vem Enhances Anti-Melanoma Efficacy *In Vitro*

**Figure 1:**
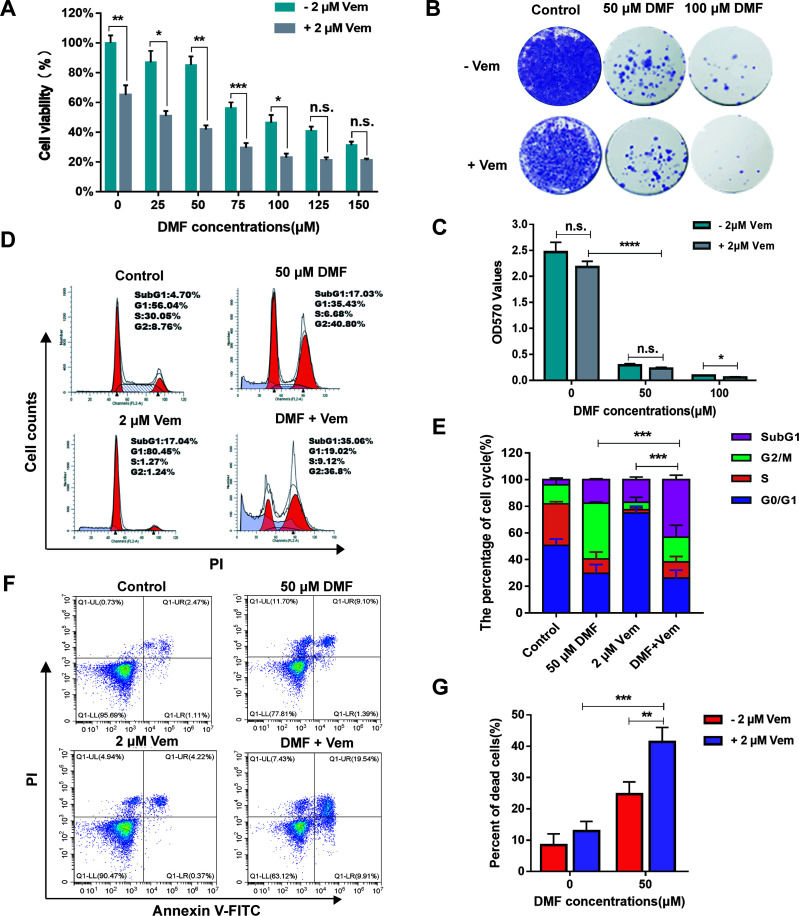
Effects of DMF/Vem on the proliferation and cell death of melanoma cells. **(A)** A375 cell viability was assessed *via* CCK-8 assay following DMF and/or Vem treatment for 48 h (n = 5), with data being shown as the percentage of absorbance at 450 nm relative to controls. **(B)** Representative images of A375 cell colonies stained with crystal violet following treatment with DMF with or without 2 μM Vem for 15 d. **(C)** Quantification of the data from **(B)**. **(D)** Representative cell cycle distribution profiles for A375 cells treated with the indicated DMF and Vem doses for 24 h followed by PI staining and flow cytometry analysis. **(E)** Quantification of the data from **(D)**. **(F)** Flow cytometry-based assessment of A375 cell death following treatment with 50 μM DMF and/or 2 μM Vem as measured by PI-Annexin V double staining. **(G)** Quantification of cell death data from **(F)**. All numerical data were from at least three independent experiments and shown as mean ± SD. *P < 0.05, **P < 0.01, ***P < 0.001, ****P < 0.0001 *vs.* DMF or Vem treatment, n.s., not significant.

To examine the ability of DMF combined with Vem to inhibit melanoma cell proliferation, we first assessed the viability of A375 melanoma cells exposed to a range of DMF concentrations (0-150 μM) alone or together with 2 μM of Vem [with this dose having been selected based on preliminary experiments and prior study (38)] for 48 h in a CCK-8 assay. Treatments of various concentrations of DMF resulted in a dose-dependent inhibition of A375 cell viability from 100.00 ± 4.98% to 31.27 ± 2.46% relative to the control cells, and treatment with 2 μM of Vem decreased the viability of these cells to 65.03 ± 6.45%, while DMF/Vem treatment reduced the viability more significantly than either DMF or Vem did (**Figure 1A**). To assess this combinational antitumor activity, we carried out colony formation assay with A375 cells treated with 50 μM and 100 μM of DMF combined with 2 μM of Vem for two-weeks. When normalized to control values, the percentage OD_570_ values revealed that Vem decreased colony formation to 87.99 ± 10.01%, while 50 μM and 100 μM of DMF decreased this activity to 11.76 ± 0.62% and 3.77 ± 0.37%, respectively, whereas DMF/Vem treatment reduced this activity to 9.26 ± 1.80% and 2.34 ± 0.75% (*P* < 0.05), respectively, at these two doses (**Figures 1B, C**). Based on the results of CCK-8 assay and colony formation assay as well as the previous studies (28, 39), we chose 50 μM of DMF for further study.

To explore the impact of DMF/Vem treatment on cell cycle progression, we employed a flow cytometry approach to assess the cell cycle distribution in those cells treated with 50 μM of DMF and/or 2 μM of Vem. The results revealed that DMF treatment increased the frequency of cells at the G2/M phase (41.87 ± 0.87%) relative to control cells (14.14 ± 4.94%) (**Figures 1D, E**), consistent with DMF induced G2/M phase arrest. Vem treatment induced G0/G1 phase arrest as evidenced by an increase in the frequency of cells in the G0/G1 phase (74.95 ± 5.18%) relative to control (50.76 ± 4.67%) (**Figures 1D, E**). DMF/Vem treatment increased the frequency of dead cells in the SubG1 phase (43.13 ± 3.35%) relative to control (3.98 ± 1.25%) (**Figures 1D, E**), consistent with combination treatment induced more cell death. We then expanded on these results by conducting a flow cytometry-based analysis of A375 cell death with Annexin V and PI dual staining. The results revealed that dead cell frequencies of 12.93 ± 3.10%, 24.70 ± 3.92%, and 41.43 ± 4.58% in the Vem, DMF, and DMF/Vem groups, respectively (**Figures 1F, G**), consistent with the enhanced induction of cell death upon combination treatment.

### Combination of DMF With Vem Improves Melanoma Therapeutic Outcomes *In Vivo*

**Figure 2:**
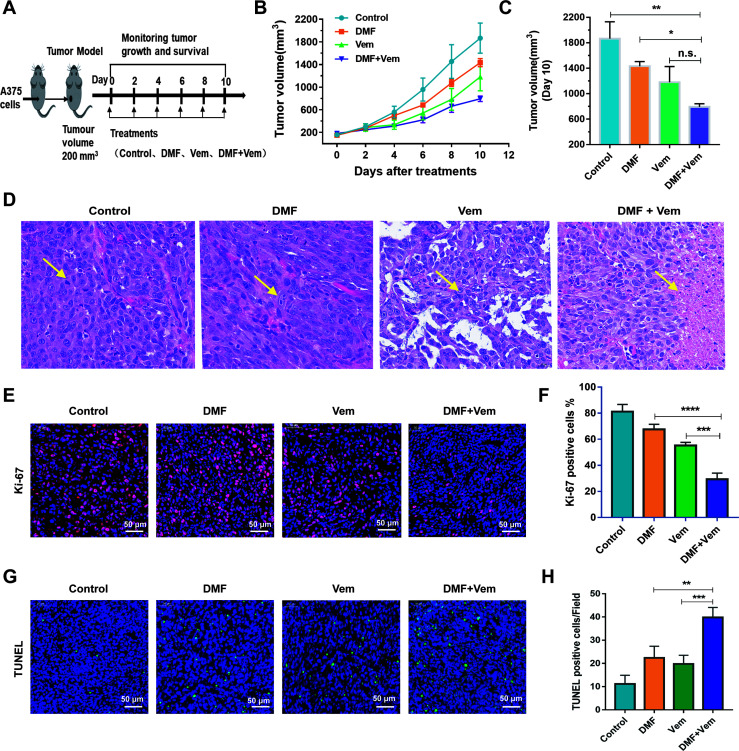
Effects of DMF/Vem treatment on melanoma *in vivo*. **(A)** Treatment strategy. A375 xenograft mice were divided into four groups (n=10): DMF treatment group (intratumoral injection of 6 mg/kg every other day), Vem treatment group (intratumoral injection of 25 mg/kg every other days), DMF/Vem treatment group (intratumoral injection of 6 mg/kg DMF and 25 mg/kg Vem every other day), and control group (DMSO-PEG400-PBS vehicle). **(B)** Tumor volumes were measured at the indicated days and calculated with the formula V= (D×d^2^)/2, (D=length, d=width). Data were expressed as mean ± SE. **(C)** Tumor volumes were measured at day 10. *P < 0.05, **P < 0.01, n.s., not significant, using one-way ANOVA. **(D)** Representative images of H&E-stained tumor sections. All images were taken at 20× magnification. **(E)** Representative images of Ki-67 IF-stained tumor sections (scale bar=50 μm). **(F)** Quantification of the data from **(E)**. ***P < 0.001 *vs.* Vem treatment, ****P < 0.0001 *vs.* DMF treatment, using one-way ANOVA. **(G)** Representative images of TUNEL-stained tumor sections (scale bar=50 μm). **(H)** Quantification of the data from **(G)**. **P < 0.01 *vs.* Vem treatment, ***P < 0.001 *vs.* DMF treatment, using one-way ANOVA.

To further expand upon these results and to better understand the antitumor efficacy of DMF/Vem treatment *in vivo*, we constructed xenograft mouse models with A375 cells and treated the mice with DMF and/or Vem by intratumoral injection through which we can precisely control the drugs dose, reduce systemic exposure of the drugs and thereby toxicity, and observe the direct effect of the drugs. We chose 6 mg/kg of DMF and 25 mg/kg of Vem (40) in our study, which correspond to the human dose given orally to psoriatic patients at 240 mg twice daily (28) and the human dose given orally to patients with BRAF-mutant melanoma at 960 mg twice daily (41) (as measured per kilogram body weight), respectively. The results showed that tumor grew slower in both single-drug treatment and combination treatment than that in control (**Figure 2B**). It was worth noting that, on day 10 of treatment, the mean tumor volume in the DMF/Vem treatment (794.00 ± 118.30 mm^3^) was smaller than that in the DMF (1432.50 ± 177.63 mm^3^) (*P* < 0.05) or Vem (1180.83 ± 604.75 mm^3^) treatment (**Figure 2C**). Treated mice did not exhibit any weight loss or other symptoms of treatment-related adverse effects during the study period (**Supplementary Figure 1**). Furthermore, the H&E staining of tumor tissue sections from these mice revealed that the tumor cells in the control group were morphologically normal with more mitotic phases, whereas cells in the Vem treatment group exhibited a reduced cytoplasmic volume and were largely mononuclear. In contrast, cells in the DMF treatment group were enlarged and some binuclear cells were evident. Notably, mitotic phases were reduced and tumor cell necrosis were increased up to 23.4 ± 4.12% (percent area) in the DMF/Vem treatment group compared with control group (**Figure 2D**). Ki-67 and terminal deoxyribonucleotidyl transferase-mediated dUTP nick end labeling (TUNEL) staining of tumor tissue sections from these mice were additionally performed to explore the effects of DMF/Vem treatment on melanoma cell proliferation and death *in vivo*. The frequencies of Ki-67 positive tumor cells in the Vem, DMF, and DMF/Vem treatments were 55.2 ± 3.19%, 67.6 ± 3.85%, and 29.4 ± 3.91%, respectively (**Figures 2E, F**). TUNEL positive (apoptotic) cells per field of view in the Vem, DMF, and DMF/Vem treatments were 19.8 ± 3.70, 22.4 ± 4.98, and 39.8 ± 4.32, respectively (**Figures 2G, H**).

### Combination of DMF With Vem Inhibits the NRF2 Antioxidant Pathway and Promotes More ROS Production

**Figure 3:**
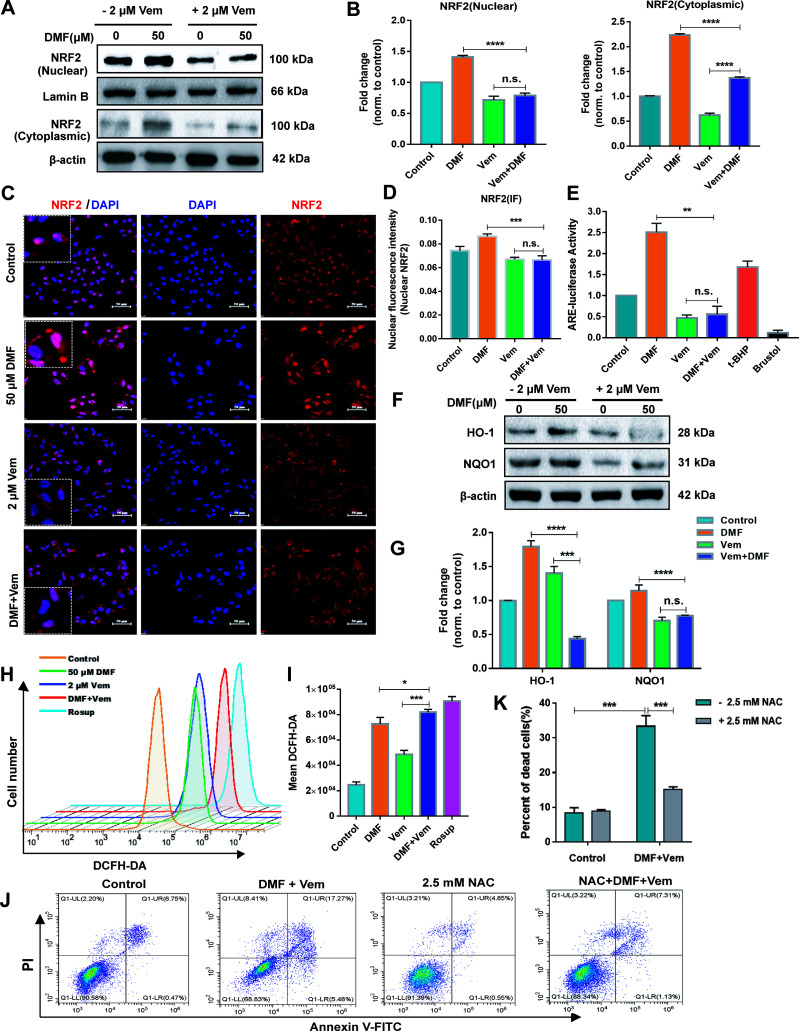
Effects of DMF/Vem treatment on NRF2 antioxidant pathway and reactive oxygen species (ROS) production in melanoma cells. **(A)** Western blot analysis of the nuclear and cytoplasmic NRF2 protein, with Lamin B or β-actin as loading control. **(B)** Protein expression in western blot **(A)** was determined by densitometry (gray value) calculations with Image J software. Data were expressed as mean ± SD (n=3). **(C)** Representative IF-staining images of NRF2 in A375 cells. Scale bar=50 μm. **(D)** Fluorescence intensity of the immunofluorescent was measured by Image-Pro-Plus 6.0 (Media Cybemetics, USA). **(E)** Quantitative and statistical analysis of ARE-luciferase activity by dual-luciferase reporter assay, with 20 μM Brusatol and 25 μM t-BHQ (tert-butyl-hydroquinone) as negative and positive controls, respectively. **(F)** Western blot analysis of HO-1 (heme oxygenase 1) and NQO1 (NAD(P)H dehydrogenase [quinone] 1) protein. **(G)** Protein expression in western blot **(F)** was determined by densitometry (gray value) calculations with Image J software. Data were expressed as mean ± SD (n=3). **(H)** Representative flow cytometry profiles of A375 cells stained by DCFH-DA, with Rosup as the positive control. **(I)** Quantitative and statistical analysis of the data from **(H)**. **(J)** Representative apoptosis profiles of A375 cells assessed *via* flow cytometry. For this assay, cells were first treated with 50 μM of DMF and 2 μM of Vem for 2 h and then exposed to 2.5 mM NAC for an additional 22 h. **(K)** Quantitative and statistical analysis of the data from **(J)**. All numerical data were from three independent experiments and shown as mean ± SD. *P < 0.05, **P < 0.01, ***P < 0.001, ****P < 0.0001 *vs.* DMF or Vem treatment, n.s., not significant, using one-way ANOVA.

To understand the mechanisms whereby DMF/Vem treatment achieved better therapeutic outcomes, we first examined the effects of these drugs on the NRF2 antioxidant pathway because DMF had previously been reported to activate this pathway and thereby exert cytoprotective and antioxidant activities in various cell types (27). Following DMF and/or Vem treatments, we assessed nuclear and cytoplasmic NRF2 levels in A375 cells. The results of western blotting showed that the nuclear and cytoplasmic NRF2 levels were higher in DMF treatment, whereas lower in Vem treatment, than those in control. Interestingly, it was less in DMF/Vem treatment than that in DMF treatment (*P* < 0.0001), but nuclear NRF2 levels in DMF/Vem treatment were insignificantly different from that in Vem treatment (n.s.), while cytoplasmic NRF2 levels in DMF/Vem treatment were higher than those in Vem treatment (*P* < 0.0001) (**Figures 3A, B**). We further confirmed these results through immunofluorescent staining. The results revealed that the fluorescence signal of NRF2 mainly located in nuclear and was stronger in DMF treatment, but weaker in Vem treatment, than that in control (**Figures 3C, D**). To evaluate the transcriptional activity of NRF2, we detected the ARE-luciferase activity driven by NRF2 with the dual-luciferase reporter gene assay and expression levels of its two target genes, *HMOX1* (heme oxygenase 1) and *NQO1* (NAD(P)H dehydrogenase [quinone] 1) with western blotting assay. The results showed that ARE-luciferase activities were higher in DMF treatment, but lower in Vem treatment, than that in control, whereas, it was lower in DMF/Vem treatment than that in DMF treatment (*P* < 0.01), and insignificantly different from that in Vem treatment (n.s.) (**Figure 3E**). The protein level of HO-1(HMOX1) was lower in DMF/Vem treatment than that in either DMF (*P* < 0.0001) or Vem (*P* < 0.001) treatment, while the protein level of NQO1 was lower in DMF/Vem treatment than that in DMF treatment (*P* < 0.0001), but insignificantly different from that in Vem treatment (n.s.) (**Figures 3F, G**).

To determine whether the effect of DMF/Vem treatment on A375 cell survival was ROS-dependent, we employed DCFH-DA fluorescence staining approach to assess ROS production in these A375 cells. Although either DMF or Vem treatment resulted in enhanced ROS levels compared to those observed in control cells, DMF/Vem treatment led to more ROS production comparing to the treatments with DMF (*P* < 0.05) or Vem (*P* < 0.001) (**Figures 3H, I**). When 2.5 mM of NAC (antioxidant N-acetyl-cysteine), a ROS inhibitor, was added into DMF/Vem treatment, cell death induced by DMF/Vem treatment was reduced (*P* < 0.001) (**Figures 3J, K**).

In addition, DMF had also been reported to decrease GSH levels and exacerbate intracellular ROS levels, ultimately inducing cell death (32). To test whether the cell death resulted from DMF treatment in our experiments was due to the GSH decrease, we measured intracellular GSH levels and cell death *via* flow cytometry after labeling cells with CMFDA and PI. The results revealed that DMF depleted intracellular GSH in a dose-dependent manner, but not all GSH-depleted cells were dead at the time of analysis (**Supplementary Figures 2A, B**).

### The Hippo and AKT/mTOR/ERK Signaling Pathways Are Altered Following Combination Treatment of DMF With Vem

**Figure 4:**
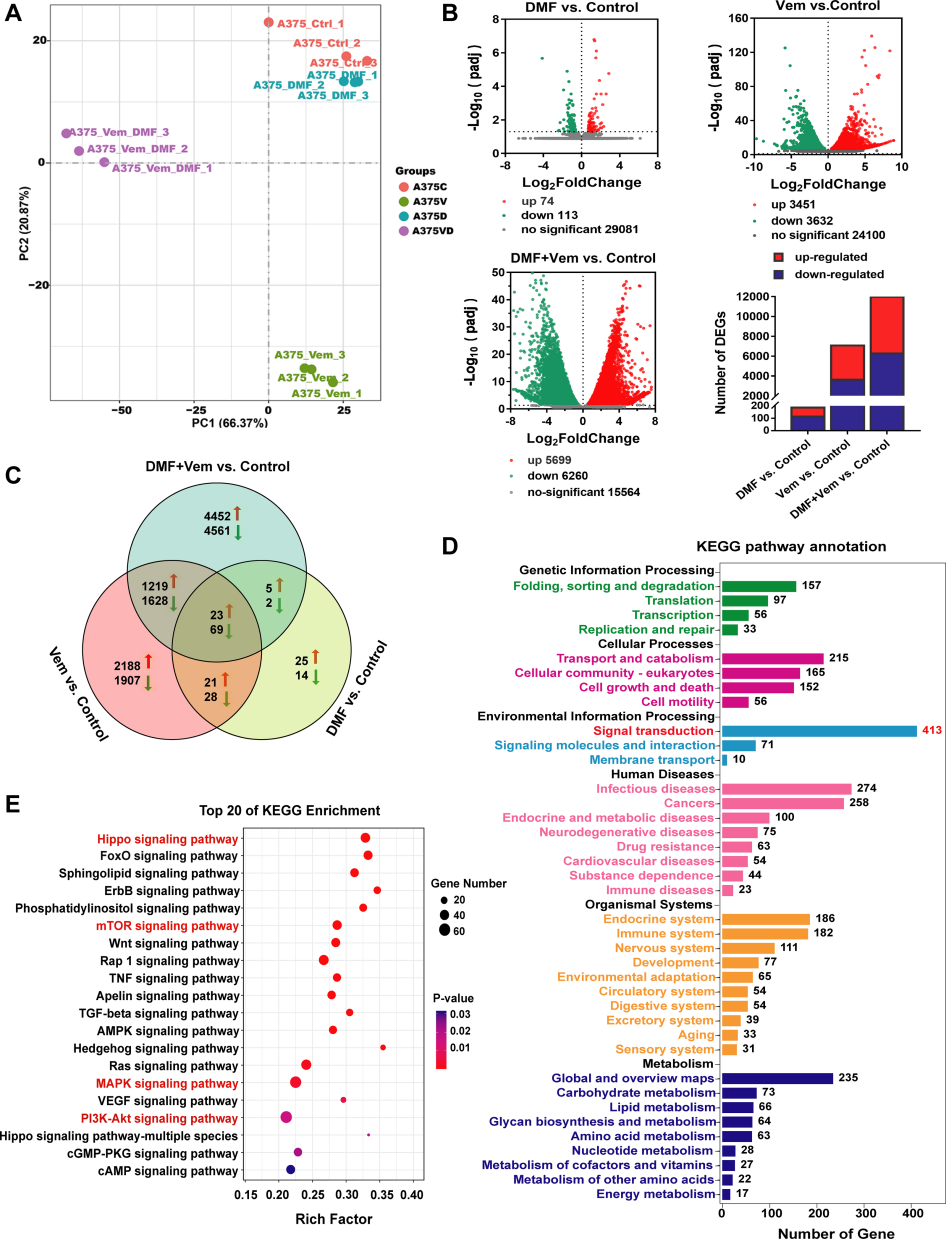
Transcriptomic analysis of A375 cells following DMF/Vem treatment. **(A)** PCA plots were used to represent A375 cell transcripts detected *via* RNA-seq following treatments with 50 μM of DMF and/or 2 μM of Vem for 24 h. **(B)** Volcano plots were used to analyze transcriptomic data, with the x-axis representing Log_2_FoldChange (sample/control) values and the y-axis representing the -Log_10_(padj). Green, red, and gray circles respectively represent genes that were downregulated, upregulated, and not differentially regulated. **(C)** Venn diagrams demonstrating the numbers of up- and down-regulated transcripts associated with each treatment. **(D)** KEGG pathway enrichment analysis of DEGs that were specifically downregulated in the context of DMF/Vem treatment. **(E)** The top 20 enriched KEGG signal transduction pathways, *P* < 0.05.

To fully explore the molecular mechanisms underlying the enhanced antitumor activity of DMF/Vem treatment, we conducted an RNA-seq analysis aiming at identifying the global transcriptomic changes in A375 cells following treatments with 50 μM of DMF and/or 2 μM of Vem for 24h, in case that intratumoral administration might lead to an uneven distribution of drug within tumor, which would affect the repeatability of samples and the accuracy of RNA-seq results. Principal component analysis (PCA) revealed that the alterations of gene expression levels were clustered in the same treatment group but obviously separated in different treatment groups (**Figure 4A**), indicating a reliable transcriptome data. Following normalization and gene filtering, 74, 3451, and 5699 up-regulated genes, while 113, 3632, and 6260 down-regulated genes were identified in DMF, Vem, and DMF/Vem treatments, respectively (**Figure 4B**). Among these differentially expressed genes (DEGs), the numbers of those in DMF/Vem treatment are much more than those in either DMF or Vem treatment.

We then assessed and arranged the DEGs in these different groups using Venn diagrams. The results revealed that 23 upregulated and 69 downregulated genes were shared among the single-drug and double-drug treatments, whereas 4452 upregulated genes and 4561 downregulated genes were observed only in the double-drug treatment (**Figure 4C**). KEGG analysis showed that the 92 shared DEGs were primarily enriched in the cell cycle and apoptosis signaling pathways (**Supplementary Figure 3A**), the 4452 upregulated genes were primarily enriched in metabolic pathways (**Supplementary Figure 3B**), while the 4561 downregulated genes were enriched in various pathways, of which 413 in signal transduction pathways (**Figure 4D**). Among the top 20 pathways (*P* < 0.05) accommodating those downregulated genes, Hippo, mTOR, MAPK, and PI3K-AKT signaling pathways (**Figure 4E**) were noteworthy, because the Hippo signaling pathway, an important regulator of the pathophysiology of cancer, had the smallest *P*-value, and the MAPK, AKT/mTOR and PI3K-AKT signaling pathways were widely believed to be the important targets for melanoma therapy.

### Combination of DMF With Vem Inhibits Hippo/YAP Pathway Activation Resulting in More Cell Death

**Figure 5:**
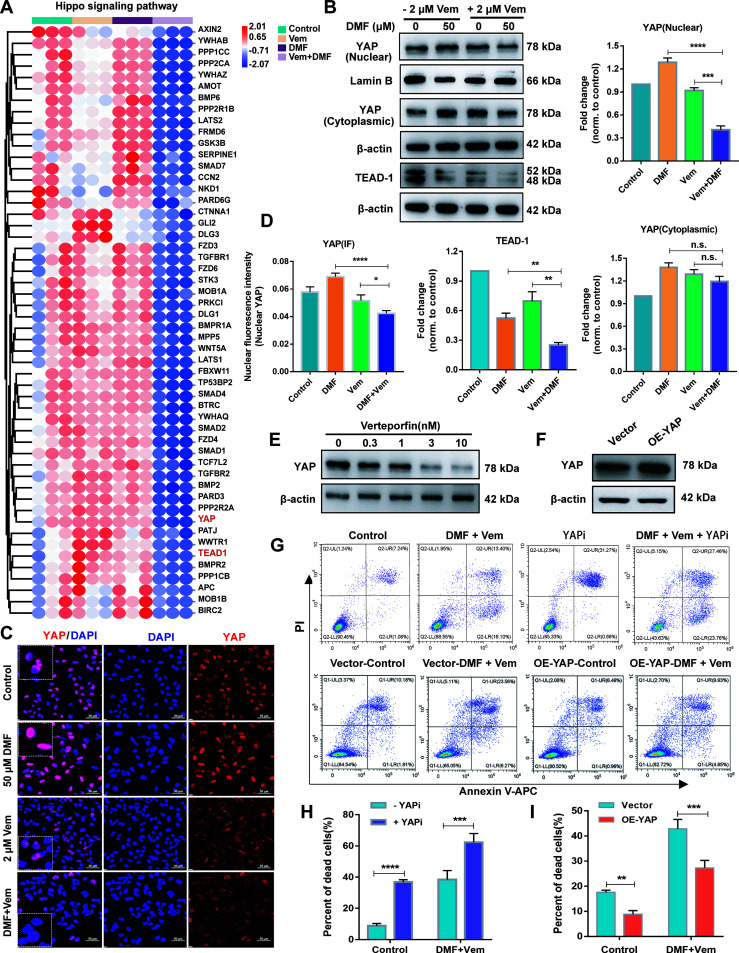
Effects of DMF/Vem treatment on Hippo pathway in melanoma cells. **(A)** The heat maps of Hippo pathway related genes downregulated upon DMF/Vem treatment. **(B)** Western blot analysis of nuclear and cytoplasmic YAP as well as TEAD-1 levels, with Lamin B or β-actin as loading control. Protein expression in western blot was determined by densitometry (gray value) calculations with Image J software. Data were expressed as mean ± SD (n=3), **P < 0.01, ***P < 0.001, ****P < 0.0001 *vs.* DMF or Vem treatment, n.s., not significant, using one-way ANOVA. **(C)** Representative YAP IF-staining images of A375 cells, scale bar=50 μm. **(D)** The fluorescence intensity of **(C)** was measured by Image-Pro-Plus 6.0 (Media Cybemetics, USA). *P < 0.05, ***P < 0.001, n.s., not significant, using one-way ANOVA. **(E)** Western blot analysis of YAP levels. For this assay, A375 cells were treated with different doses of YAPi (verteporfin) for 24 h. **(F)** Western blot analysis of YAP overexpression levels. A375 cells were transfected with YAP overexpression vector for 72 h. **(G)** Representative flow cytometry profiles of A375 cells stained with Annexin V and PI. *Top*: Flow cytometry was used to assess cell death of A375 treated with DMF/Vem or 3 nM verteporfin plus DMF/Vem for 24 h. *Bottom*: A375 cells were transfected with YAP overexpression vector for 48 h, then flow cytometry was used to assess the cell death after treatment with 50 μM of DMF plus 2 μM of Vem for 24 h. **(H)** Quantitative and statistical analysis of the data from (F: *Top*). Data were shown as mean ± SD (n=3), ***P < 0.001, ****P < 0.0001, using two-way ANOVA to compare the differences between the groups. **(I)** Quantitative and statistical analysis of the data from (F: *Bottom*). Data were shown as mean ± SD (n=3), **P < 0.01, using two-way ANOVA to compare the differences between the groups.

To demonstrate the role of Hippo signaling pathway may play in the enhanced anti-melanoma effects of DMF/Vem treatment, we extracted 52 Hippo pathway related genes from those downregulated genes. The heat maps compiling of the 52 downregulated DEGs revealed that expressions of these genes were substantially inhibited in DMF/Vem treatment compared to those in DMF or Vem treatment (**Figure 5A**). Among the 52 DEGs, the expression of *YAP* and *TEAD-1*, two downstream Hippo effector genes, were further verified by qRT-PCR and western blotting. The significant decreases of *YAP* and *TEAD-1* mRNA levels were confirmed in DMF/Vem treatment, whereas the significant increases were observed in either DMF or Vem treatments (**Supplementary Figure 4**). The nuclear protein levels of YAP were lower in DMF/Vem treatment than those in DMF (*P* < 0.0001) or Vem (*P* < 0.001) treatment, whereas the cytoplasmic levels were about the same among DMF/Vem treatment and single-drug treatments (n.s.) (**Figure 5B**). Immunofluorescent staining further confirmed that the YAP level in DMF/Vem treatment was lower than that in either DMF or Vem treatments (**Figures 5C, D**). The immunohistochemical staining results of YAP in tumor sections also showed that YAP expression in DMF/Vem treatment was lower than that in DMF (*P* < 0.0001) or Vem (*P* < 0.001) treatment (**Supplementary Figure 5**). The results of western blotting also showed that TEAD-1 protein levels were much lower in DMF/Vem treatment than those in either DMF (*P* < 0.01) or Vem (*P* < 0.01) treatment (**Figure 5B**).

Next, we inhibited YAP with verteporfin (a YAP inhibitor, YAPi) (**Figure 5E**) or overexpressed YAP (**Figure 5F**) by transfection of a YAP overexpression vector in A375 cells to assess the cell death following DMF/Vem treatment under the conditions of altered YAP expression or activity. The results showed that the frequency of total dead cells was higher in YAPi/DMF/Vem treatment than that in DMF/Vem treatment (*P* < 0.001) (**Figures 5G, H**), whereas it was lower in YAP-overexpressing A375 cells than that in the empty vector transfected A375 cells following DMF/Vem treatment (*P* < 0.01) (**Figures 5G, I**).

### Combination of DMF With Vem Downregulates MAPK and AKT/mTOR Pathways Related Proteins Expression

**Figure 6:**
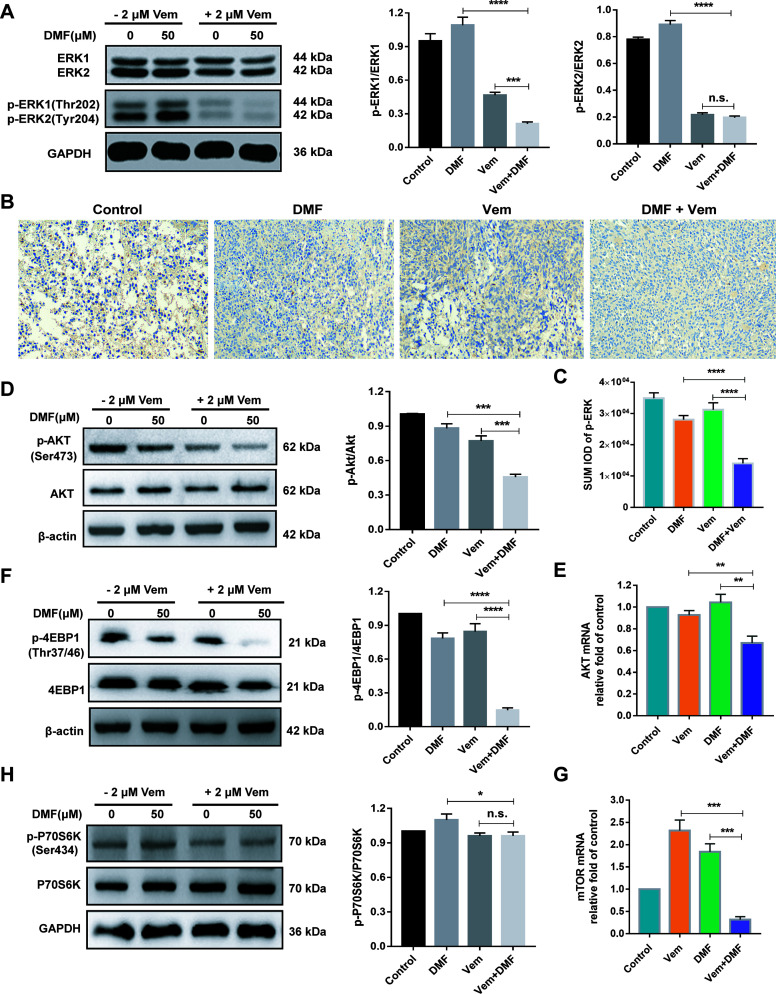
Effects of DMF/Vem treatment on MAPK and AKT/mTOR pathways related proteins in melanoma cells. **(A)** Western blot analysis of total and phosphorylated ERK1/2 in A375 cells treated with 50 μM of DMF and/or 2 μM of Vem for 48 h, with GAPDH as loading control. **(B)** Representative images of p-ERK immunohistochemical staining in tumor tissue. All images were taken at 10× magnification. **(C)** The integrated optical density (IOD) of immunohistochemical results of p-ERK was calculated by Image Pro Plus 6.0. ****P < 0.0001, using one-way ANOVA to compare the differences between the groups. **(D)** Western blot analysis of total and phosphorylated AKT in A375 cells treated with 50 μM of DMF and/or 2 μM of Vem for 48 h. **(E)** qPCR analysis of *AKT1* mRNA levels in A375 cells following treatments with 50 μM of DMF and/or 2 μM of Vem for 24 h, with *18S* as reference control. **(F)** Western blot analysis of total and phosphorylated 4EBP1 in A375 cells treated with 50 μM of DMF and/or 2 μM of Vem for 48 h, with GAPDH as loading control. **(G)** qPCR analysis of *mTOR* mRNA levels in A375 cells treated with 50 μM of DMF and/or 2 μM of Vem for 24 h, with *18S* as reference control. **(H)** Western blot analysis of total and phosphorylated P70S6K in A375 cells treated with 50 μM of DMF and/or 2 μM of Vem for 48 h, with GAPDH as loading control. Protein expression in western blot was determined by densitometry (gray value) calculations with Image J software. All numerical data were shown as mean ± SD (n=3), *P < 0.05, **P < 0.01, ***P < 0.001, ****P < 0.0001 *vs.* DMF or Vem treatment, n.s., not significant, using one-way ANOVA to compare the differences between the groups.

The RNA-seq results mentioned above indicated a potential relationship between the enhanced antitumor activity of DMF/Vem treatment and the MAPK, PI3K-AKT and mTOR signaling pathways. To confirm this relationship, we first assessed phosphorylated and total ERK1/2 levels *via* western blotting. The results showed p-ERK1 level in DMF/Vem treatment was lower than that in DMF (*P* < 0.0001) or Vem (*P* < 0.001) treatment, whereas p-ERK2 level was much lower in DMF/Vem treatment than that in DMF treatment (*P* < 0.0001), but was about the same as that in Vem treatment (n.s.) (**Figure 6A**). The immunohistochemical staining results for p-ERK in tumor sections also showed that p-ERK expression in DMF/Vem treatment was lower than that in DMF (*P* < 0.0001) or Vem (*P* < 0.0001) treatment (**Figures 6B, C**). We then evaluated mRNA and protein levels of AKT by qRT-PCR and western blotting, respectively. The results showed that both mRNA and phosphorylated levels of AKT in DMF/Vem treatment were much lower than those in DMF (mRNA: *P* < 0.01, phosphorylated levels: *P* < 0.001) or Vem (mRNA: *P* < 0.01, phosphorylated levels: *P* < 0.001) treatment (**Figures 6D, E**). Finally, we detected the mRNA level of mTOR and protein levels of 4EBP1 and P70S6K, which are two downstream mTOR signaling cascade effectors. The results revealed that mRNA level of mTOR and protein level of p-4EBP1 were lower in DMF/Vem treatment than those in single-agent treatments (*mTOR*: *P* < 0.001, p-4EBP1: *P* < 0.0001) (**Figures 6F, G**), whereas the protein level of p-P70S6K was lower in DMF/Vem treatment than that in DMF treatment (*P* < 0.05), but was about the same as that in Vem treatment (n.s.) (**Figure 6H**).

## Discussion

DMF had been reported to inhibit A375 cell proliferation in a dose-dependent manner and induce G2/M phase arrest as well as apoptosis, while Vem induced G0/G1 phase arrest and less pronounced apoptotic cell death (28, 42). Our results (**Figures 1A, D–G**) confirmed those previous achievements. DMF was used in combination with DTIC as a new therapeutic option for treatment of the metastatic melanoma. DTIC/DMF treatment impaired melanoma cell migration *in vitro* and slightly reduced melanoma volumes in mouse model. But the additive effects of DTIC and DMF on cell proliferation or apoptosis were not identified (35). Here, we found DMF/Vem treatment impaired melanoma cell proliferation (**Figure 1A** and **Figures 2E, F**) and colony formation (**Figures 1B, C**) much more sufficiently, and induced more tumor cell death (**Figures 1F, G** and **Figures 2G, H**) than the treatments with either DMF or Vem alone *in vitro* and *in vivo*. We postulated that the apoptosis-inducing abilities of DMF and Vem *via* cell cycle arrest at different cell phases could overlap to lead to an overwhelmingly tumor-killing effect, highlighting a promising benefit of the DMF/Vem-based treatment for melanoma.

A growing body of research evidences indicated that DMF could exert cytoprotective and antioxidant effects in several noncancer cell models (27) and ovarian cancer cells (32), primarily through the activation of the NRF2 antioxidant pathway (43). NRF2 functions as a leucine zipper transcription factor responsible for protecting cells against oxidative stress and associated ROS-related damage. Under homeostatic conditions, NRF2 is sequestered in the cytoplasm by Kelch-like ECH-associated protein 1 (Keap1), which promotes its degradation (44). DMF can promote the succination of two key cysteine residues in Keap1, thereby disrupting the interactions between Keap1 and NRF2, release NRF2 to undergo nuclear translocation (45). In the nucleus, NRF2 can subsequently bind to the antioxidant response elements (AREs) and promote expression of target genes including *HMOX1* and *NQO1*, which generate the major cellular antioxidant GSH (46, 47). It was reported that high-dose DMF (100 μM) did not alter NRF2 and HO-1(HMOX1) levels in ovarian carcinoma cells OVCAR3, but induced parallel increase of oxidative stress and GSH depletion, thus displayed cytotoxicity, whereas, lower dose DMF (0.25-5 μM) induced NRF2 activation and were cytoprotective (32). Here our results that 50 μM of DMF induced GSH depletion (**Supplementary Figure 2**) and increased expression and nuclear translocation of NRF2 as well as the protein levels of HO-1 and NQO1 in melanoma cells (**Figures 3A–G**) were consistent with the previous observations (32), suggesting DMF have similar functions in melanoma as in other cancer lines.

It sounds contradictory that DMF treatment could induce both GSH depletion which has cytotoxicity and NRF2 activation which should display a cytoprotective effect. However, DMF had also been reported to suppress the activity of γ‐GCS, which is the first enzyme in the GSH biosynthesis pathway (32), thereby impairing GSH production and exacerbating ROS-induced cellular damage, culminating in cellular death in several cancer cell lines. Our results confirmed that DMF treatment enhanced ROS generation (**Figures 3H, I**). Therefore, DMF seemed to have a dualistic effect on cell survival through activating NRF2 and promoting ROS. But, when combined with Vem, DMF/Vem treatment only exhibited an enhanced tumor-killing effect in our experiments (**Figures 1F, G**, **Figures 2G, H**, **Figures 3A–I**). Thus, Vem should have interrupted the cytoprotective function of DMF and/or simultaneously reinforced its cell-killing function.

Vem was reported to inhibit NRF2 activation while enhance mitochondrial respiration and ROS production in melanoma cells (48, 49). Our data also confirmed that Vem significantly reduced the expression and transcriptional activity of NRF2 and enhanced ROS production in melanoma cells (**Figures 3A–I**). Moreover, DMF/Vem treatment induced more robust ROS production (**Figures 3H, I**) and more pronounced cell death (**Figures 1F, G** and **Figures 2G, H**) than either Vem or DMF treatment did. This meant that Vem impaired the ability of DMF to activate NRF2, thereby reduced GSH production, while bolstered up ROS generation, resulting in a boost of overall ROS and more cell death. To verify this speculation, we added NAC, an antioxidant which had been reported to both suppress the generation and damage the activity of ROS within various cells (50), into the DMF/Vem treatment to evaluate the alteration of cell death. Our results that NAC treatment sufficiently prevented melanoma cell from death induced by DMF/Vem treatment (**Figures 3J, K**) confirmed that the cell death induced by DMF/Vem at least in part through the induction of a more robust burst of ROS production. The oxidative stress was widely believed to disrupt the mitochondrial functionality and thereby engage in apoptotic signaling cascades. Augmenting ROS levels within tumor cells may represent an effective anticancer treatment strategy (51). The observation of enhancement of ROS production in melanoma cells following DMF/Vem treatment (**Figures 3H, I**), suggested that this combination treatment may become a promising therapeutic regimen for melanoma.

ROS level is balanced by that of GSH which is largely determined by NRF2-ARE pathway. DMF/Vem treatment inhibited nuclear NRF2 expression and its transcriptional activity less significantly than Vem treatment did in our experiments (**Figures 3A–G**) but induced more melanoma cell death than Vem treatment did *in vitro* (**Figures 1F, G**) and *in vivo* (**Figures 2G, H**). This implied the extra cell death induced by DMF/Vem treatment might result from the mechanisms other than the inhibition of NRF2-ARE pathway. To globally explore the unknown mechanisms underlying the extra cell death induced by DMF/Vem treatment, we conducted an RNA-seq analysis to distinguish the DEGs specifically regulated by DMF/Vem treatment using A375 cells in case of the cell heterogeneity in tumor tissue. The results showed the number of DEGs affected by DMF/Vem treatment was far more than that induced by either DMF or Vem treatment (**Figure 4B**), and the number of DEGs specifically affected by DMF/Vem treatment was also much more than that of the common DEGs in DMF and/or Vem treatments (**Figure 4C**). This suggested DMF and Vem could collaborate to interrupt much more gene expressions other than single-drug treatments did. Moreover, those DMF/Vem-specific DEGs were mainly enriched in signal transduction pathways, especially in the Hippo signaling pathway.

Hippo signaling pathway is an important regulator of the pathophysiology of cancer, regulating important malignant cellular processes including survival, proliferation, metastasis, and cell fate determination (52). YAP functions as a downstream effector protein in the Hippo signaling pathway (52). Its activity is regulated by its rate of transit between the nuclear and cytoplasmic compartments (53, 54). Nuclear localization of YAP is believed to be a poor prognosis in patients with cancer (55). Inhibiting the expression of YAP was reported to suppress cellular proliferation and induce pronounced cell death by modulating the transcription of downstream target genes including the transcription factors *TEAD1*-*TEAD4* (55, 56). Our data revealed that both expression and nuclear translocation of YAP and its target gene *TEAD-1* were suppressed in DMF/Vem treatment, but not in either DMF or Vem treatment (**Figures 5A–D**), suggesting the extra cell death in DMF/Vem treatment may be a result of Hippo signaling pathway interruption. This suggestion was further supported by the evidence that the frequency of dead cell in DMF/Vem treatment was increased by YAPi treatment (**Figures 5E, G, H**) while decreased by the overexpression of YAP (**Figures 5F, G, I**), confirming YAP inhibition was one of the mechanisms underlying the extra cell death induced by DMF/Vem treatment. In addition, YAP was also observed to play an important role in regulating the sensitivity of *BRAF* and *KRAS* mutant cancer cells to specific drugs (57–59), and the invasive activity of melanoma *in vitro* and *in vivo* (60). Thus YAP may be a promising target for melanoma treatment.

Moreover, it is well known that MAPK and AKT/mTOR pathways play well-documented roles in modulating cell growth, proliferation and survival, and form a series of regulatory feedback loops. Excess AKT/mTOR pathway activation had been identified as a facilitator of melanoma cell survival in the context of *BRAF^V600E^* inhibition (61–63). To overcome the drug acquired resistance of melanoma, combination treatments of BRAFi with MEKi had become the default therapies for patients with advanced BRAF^V600^-mutated melanoma. Vem/cobimetinib treatment was reported to inhibit cell proliferation in melanoma cells (64, 65), and abrogate the paradoxical activation of the MAPK pathway and inhibit glucose metabolism pathway (66), but no inhibition of other pathways was reported. In this study, DMF/Vem treatment suppressed the phosphorylation of ERK1, AKT and 4EBP1 more effectively than either DMF or Vem treatment did (**Figures 6A–F**), indicating DMF/Vem treatment simultaneously inhibited the activation of MAPK and AKT/mTOR signaling pathways. Apart from the Hippo, MAPK and AKT/mTOR pathways, our transcriptome analysis also showed that dozens of other signaling pathways (**Figure 4E**) and metabolism pathways (**Figure 4D**) were suppressed by DMF/Vem treatment. This suggested that simultaneous inhibition of multiple signaling pathways should also be a mechanism underlying the extra cell death induced by DMF/Vem treatment.

It is worth to note that there is nonetheless a clear need for future in-depth analyses of the pharmacokinetic properties and safety profiles associated with systemic DMF/Vem treatment in order to fully understand the benefits and potential risks associated with such a therapeutic approach, as DMF and Vem are both clinically oral drugs in patients. We plan to conduct patient-derived xenograft (PDX) models to further evaluate the therapeutic efficacy and toxicity of this combination treatment regimen in the near future.

In conclusion, our results for the first time demonstrated that DMF/Vem treatment significantly enhanced suppression of melanoma cell proliferation and increased tumor cell death *in vitro* and *in vivo*. These anticancer activities were at least partially attributable to the induction of a burst of reactive oxygen species (ROS) production and concomitant inhibition of the Hippo/YAP, NRF2-ARE, and AKT/mTOR/ERK signaling pathways. The combination approach of DMF with Vem proposed here would be a novel promising therapeutic strategy for melanoma.

## Data Availability Statement

The datasets presented in this study can be found in online repositories. The names of the repository/repositories and accession number(s) is the NCBI Sequence Read Archive and PRJNA769791, respectively.

## Ethics Statement

The animal study was reviewed and approved by The Ethics Committee of the Inner Mongolia University.

## Author Contributions

HL, JQ, and WX conceived and designed the study. HL, YPW, RS, and XL performed the experiments *in vitro*. HL, RS, YWW, and YF performed the experiments *in vivo*. HL, YJ, and HS analysed the results. HL wrote the manuscript. WX polished the language. All authors contributed to the article and approved the submitted version.

## Funding

This study was supported in part by the National Natural Science Foundation of China (Grant Number: 32060173 and 31460600), and Natural Science Foundation of Inner Mongolia (Grant Number: 2020MS08098, 2019MS03027, and 2018MS08027).

## Conflict of Interest

The authors declare that the research was conducted in the absence of any commercial or financial relationships that could be construed as a potential conflict of interest.

## Publisher’s Note

All claims expressed in this article are solely those of the authors and do not necessarily represent those of their affiliated organizations, or those of the publisher, the editors and the reviewers. Any product that may be evaluated in this article, or claim that may be made by its manufacturer, is not guaranteed or endorsed by the publisher.

## References

[1] Gray-Schopfer V Wellbrock C Marais R. Melanoma Biology and New Targeted Therapy. Nature (2007) 445(7130):851–7. doi: 10.1038/nature05661 17314971

[2] Shain AH Bastian BC. From Melanocytes to Melanomas. Nat Rev Cancer (2016) 16(6):345–58. doi: 10.1038/nrc.2016.37 27125352

[3] Strickland LR Pal HC Elmets CA Afaq F. Targeting Drivers of Melanoma With Synthetic Small Molecules and Phytochemicals. Cancer Lett (2015) 359(1):20–35. doi: 10.1016/j.canlet.2015.01.016 25597784 PMC4599342

[4] Franzke A Probst-Kepper M Buer J Duensing S Hoffmann R Wittke F. Elevated Pretreatment Serum Levels of Soluble Vascular Cell Adhesion Molecule 1 and Lactate Dehydrogenase as Predictors of Survival in Cutaneous Metastatic Malignant Melanoma. Br J Cancer (1998) 78(1):40–5. doi: 10.1038/bjc.1998.439 PMC2062938 9662248

[5] Bhatia S Tykodi SS Thompson JA. Treatment of Metastatic Melanoma: An Overview. Oncol (Williston Park) (2009) 23(6):488–96. PMC2737459 19544689

[6] Domingues B Lopes JM Soares P Populo H. Melanoma Treatment in Review. Immunotargets Ther (2018) 7:35–49. doi: 10.2147/ITT.S134842 29922629 PMC5995433

[7] Kozar I Margue C Rothengatter S Haan C Kreis S. Many Ways to Resistance: How Melanoma Cells Evade Targeted Therapies. Biochim Biophys Acta Rev Cancer (2019) 1871(2):313–22. doi: 10.1016/j.bbcan.2019.02.002 30776401

[8] Eggermont AMM Spatz A Robert C. Cutaneous Melanoma. Lancet (2014) 383(9919):816–27. doi: 10.1016/s0140-6736(13)60802-8 24054424

[9] Grossman D Altieri DC. Drug Resistance in Melanoma: Mechanisms, Apoptosis, and New Potential Therapeutic Targets. Cancer Metastasis Rev (2001) 20(1-2):3–11. doi: 10.1023/a:1013123532723 11831644

[10] Hodis E Watson IR Kryukov GV Arold ST Imielinski M Theurillat JP. A Landscape of Driver Mutations in Melanoma. Cell (2012) 150(2):251–63. doi: 10.1016/j.cell.2012.06.024 PMC3600117 22817889

[11] Cancer Genome Atlas N. Genomic Classification of Cutaneous Melanoma. Cell (2015) 161(7):1681–96. doi: 10.1016/j.cell.2015.05.044 PMC4580370 26091043

[12] Jenkins RW Fisher DE. Treatment of Advanced Melanoma in 2020 and Beyond. J Invest Dermatol (2021) 141(1):23–31. doi: 10.1016/j.jid.2020.03.943 32268150 PMC7541692

[13] Hauschild A Ascierto PA Schadendorf D Grob JJ Ribas A Kiecker F. Long-Term Outcomes in Patients With BRAF V600-Mutant Metastatic Melanoma Receiving Dabrafenib Monotherapy: Analysis From Phase 2 and 3 Clinical Trials. Eur J Cancer (2020) 125:114–20. doi: 10.1016/j.ejca.2019.10.033 PMC8073226 31864178

[14] Luo C Shen J. Research Progress in Advanced Melanoma. Cancer Lett (2017) 397:120–6. doi: 10.1016/j.canlet.2017.03.037 28385603

[15] Chapman PB Hauschild A Robert C Haanen JB Ascierto P Larkin J. Improved Survival With Vemurafenib in Melanoma With BRAF V600E Mutation. N Engl J Med (2011) 364(26):2507–16. doi: 10.1056/NEJMoa1103782 PMC3549296 21639808

[16] Moriceau G Hugo W Hong A Shi H Kong X Yu CC. Tunable-Combinatorial Mechanisms of Acquired Resistance Limit the Efficacy of BRAF/MEK Cotargeting But Result in Melanoma Drug Addiction. Cancer Cell (2015) 27(2):240–56. doi: 10.1016/j.ccell.2014.11.018 PMC4326539 25600339

[17] Dietrich P Kuphal S Spruss T Hellerbrand C Bosserhoff AK. Wild-Type KRAS is a Novel Therapeutic Target for Melanoma Contributing to Primary and Acquired Resistance to BRAF Inhibition. Oncogene (2018) 37(7):897–911. doi: 10.1038/onc.2017.391 29059159

[18] Niezgoda A Niezgoda P Czajkowski R. Novel Approaches to Treatment of Advanced Melanoma: A Review on Targeted Therapy and Immunotherapy. BioMed Res Int (2015) 2015:851387. doi: 10.1155/2015/851387 26171394 PMC4478296

[19] Dummer R Ascierto PA Gogas HJ Arance A Mandala M Liszkay G. Overall Survival in Patients With BRAF-Mutant Melanoma Receiving Encorafenib Plus Binimetinib Versus Vemurafenib or Encorafenib (COLUMBUS): A Multicentre, Open-Label, Randomised, Phase 3 Trial. Lancet Oncol (2018) 19(10):1315–27. doi: 10.1016/s1470-2045(18)30497-2 30219628

[20] Robert C Karaszewska B Schachter J Rutkowski P Mackiewicz A Stroiakovski D. Improved Overall Survival in Melanoma With Combined Dabrafenib and Trametinib. N Engl J Med (2015) 372(1):30–9. doi: 10.1056/NEJMoa1412690 25399551

[21] Larkin J Ascierto PA Dreno B Atkinson V Liszkay G Maio M. Combined Vemurafenib and Cobimetinib in BRAF-Mutated Melanoma. N Engl J Med (2014) 371(20):1867–76. doi: 10.1056/NEJMoa1408868 25265494

[22] Long GV FK Stroyakovskiy D Gogas H Levchenko E de Braud F Larkin J. Dabrafenib Plus Trametinib Versus Dabrafenib Monotherapy in Patients With Metastatic BRAF V600EK–Mutant Melanoma Long-Term Survival and Safety Analysis of a Phase 3 Study. Ann Oncol (2017) 28(7):1631–9. doi: 10.1093/annonc/mdx176 PMC5834102 28475671

[23] Robert C Grob JJ Stroyakovskiy D Karaszewska B Hauschild A Levchenko E. Five-Year Outcomes With Dabrafenib Plus Trametinib in Metastatic Melanoma. N Engl J Med (2019) 381(7):626–36. doi: 10.1056/NEJMoa1904059 31166680

[24] Brighton HE Angus SP Bo T Roques J Tagliatela AC Darr DB. New Mechanisms of Resistance to MEK Inhibitors in Melanoma Revealed by Intravital Imaging. Cancer Res (2018) 78(2):542–57. doi: 10.1158/0008-5472.CAN-17-1653 PMC6132242 29180473

[25] Patel H Yacoub N Mishra R White A Long Y Alanazi S. Current Advances in the Treatment of BRAF-Mutant Melanoma. Cancers (Basel) (2020) 12(2):482. doi: 10.3390/cancers12020482 PMC7072236 32092958

[26] Hao M Song F Du X Wang G Yang Y Chen K. Advances in Targeted Therapy for Unresectable Melanoma: New Drugs and Combinations. Cancer Lett (2015) 359(1):1–8. doi: 10.1016/j.canlet.2014.12.050 25578781

[27] Saidu NEB Kavian N Leroy K Jacob C Nicco C Batteux F. Dimethyl Fumarate, a Two-Edged Drug: Current Status and Future Directions. Med Res Rev (2019) 39(5):1923–52. doi: 10.1002/med.21567 30756407

[28] Loewe R Valero T Kremling S Pratscher B Kunstfeld R Pehamberger H. Dimethylfumarate Impairs Melanoma Growth and Metastasis. Cancer Res (2006) 66(24):11888–96. doi: 10.1158/0008-5472.CAN-06-2397 17178886

[29] Yamazoe Y Tsubaki M Matsuoka H Satou T Itoh T Kusunoki T. Dimethylfumarate Inhibits Tumor Cell Invasion and Metastasis by Suppressing the Expression and Activities of Matrix Metalloproteinases in Melanoma Cells. Cell Biol Int (2009) 33(10):1087–94. doi: 10.1016/j.cellbi.2009.06.027 19595779

[30] Takeda T Tsubaki M Asano R Itoh T Imano M Satou T. Dimethyl Fumarate Suppresses Metastasis and Growth of Melanoma Cells by Inhibiting the Nuclear Translocation of NF-kappaB. J Dermatol Sci (2020) 99(3):168–76. doi: 10.1016/j.jdermsci.2020.07.004 32693971

[31] Kastrati I Siklos MI Calderon-Gierszal EL El-Shennawy L Georgieva G Thayer EN. Dimethyl Fumarate Inhibits the Nuclear Factor kappaB Pathway in Breast Cancer Cells by Covalent Modification of P65 Protein. J Biol Chem (2016) 291(7):3639–47. doi: 10.1074/jbc.M115.679704 PMC4751401 26683377

[32] Saidu NE Noe G Cerles O Cabel L Kavian-Tessler N Chouzenoux S. Dimethyl Fumarate Controls the NRF2/DJ-1 Axis in Cancer Cells: Therapeutic Applications. Mol Cancer Ther (2017) 16(3):529–39. doi: 10.1158/1535-7163.MCT-16-0405 28069874

[33] Xie X Zhao Y Ma C-Y Xu X-M Zhang Y-Q Wang C-G. Dimethyl Fumarate Induces Necroptosis in Colon Cancer Cells Through GSH Depletion/ROS Increase/MAPKs Activation Pathway. Br J Pharmacol (2015) 172(15):3929–43. doi: 10.1111/bph.13184 PMC4523346 25953698

[34] Tavallai M Booth L Roberts JL McGuire WP Poklepovic A Dent P. Ruxolitinib Synergizes With DMF to Kill *via* BIM Plus BAD-Induced Mitochondrial Dysfunction and *via* Reduced SOD2/TRX Expression and ROS. Oncotarget (2016) 7(14):17290–300. doi: 10.18632/oncotarget.8039 PMC4951212 26981780

[35] Valero T Steele S Neumuller K Bracher A Niederleithner H Pehamberger H. Combination of Dacarbazine and Dimethylfumarate Efficiently Reduces Melanoma Lymph Node Metastasis. J Invest Dermatol (2010) 130(4):1087–94. doi: 10.1038/jid.2009.368 19940857

[36] Jaykaran Charan NDK. How to Calculate Sample Size in Animal Studies. J Pharmacol Pharmacotherapeut (2013) 4(4):303–6. doi: 10.4103/0976-500X.119726 PMC3826013 24250214

[37] Du P Ma Q Zhu ZD Li G Wang Y Li QQ. Mechanism of Corilagin Interference With IL-13/STAT6 Signaling Pathways in Hepatic Alternative Activation Macrophages in Schistosomiasis-Induced Liver Fibrosis in Mouse Model. Eur J Pharmacol (2016) 793:119–26. doi: 10.1016/j.ejphar.2016.11.018 27845069

[38] Wang L Leite de Oliveira R Huijberts S Bosdriesz E Pencheva N Brunen D. An Acquired Vulnerability of Drug-Resistant Melanoma With Therapeutic Potential. Cell (2018) 173(6):1413–25.e14. doi: 10.1016/j.cell.2018.04.012 29754815

[39] Kaluzki I Hrgovic I Hailemariam-Jahn T Doll M Kleemann J Valesky EM. Dimethylfumarate Inhibits Melanoma Cell Proliferation *via* P21 and P53 Induction and Bcl-2 and Cyclin B1 Downregulation. Tumour Biol (2016) 37(10):13627–35. doi: 10.1007/s13277-016-5285-6 27468725

[40] Yang H Higgins B Kolinsky K Packman K Go Z Iyer R. RG7204 (PLX4032), A Selective BRAFV600E Inhibitor, Displays Potent Antitumor Activity in Preclinical Melanoma Models. Cancer Res (2010) 70(13):5518–27. doi: 10.1158/0008-5472.CAN-10-0646 20551065

[41] Bollag G Hirth P Tsai J Zhang J Ibrahim PN Cho H. Clinical Efficacy of a RAF Inhibitor Needs Broad Target Blockade in BRAF-Mutant Melanoma. Nature (2010) 467(7315):596–9. doi: 10.1038/nature09454 PMC2948082 20823850

[42] Sondergaard JN Nazarian R Wang Q Guo D Hsueh T Mok S. Differential Sensitivity of Melanoma Cell Lines With BRAFV600E Mutation to the Specific Raf Inhibitor PLX4032. J Transl Med (2010) 8:39. doi: 10.1186/1479-5876-8-39 20406486 PMC2876068

[43] Scannevin RH Chollate S Jung MY Shackett M Patel H Bista P. Fumarates Promote Cytoprotection of Central Nervous System Cells Against Oxidative Stress *via* the Nuclear Factor (Erythroid-Derived 2)-Like 2 Pathway. J Pharmacol Exp Ther (2012) 341(1):274–84. doi: 10.1124/jpet.111.190132 22267202

[44] Wakabayashi N Itoh K Wakabayashi J Motohashi H Noda S Takahashi S. Keap1-Null Mutation Leads to Postnatal Lethality Due to Constitutive Nrf2 Activation. Nat Genet (2003) 35(3):238–45. doi: 10.1038/ng1248 14517554

[45] Yang M Soga T Pollard PJ Adam J. The Emerging Role of Fumarate as an Oncometabolite. Front Oncol (2012) 2:85. doi: 10.3389/fonc.2012.00085 22866264 PMC3408580

[46] Konstantinopoulos PA Spentzos D Fountzilas E Francoeur N Sanisetty S Grammatikos AP. Keap1 Mutations and Nrf2 Pathway Activation in Epithelial Ovarian Cancer. Cancer Res (2011) 71(15):5081–9. doi: 10.1158/0008-5472.CAN-10-4668 21676886

[47] Itoh K Tong KI Yamamoto M. Molecular Mechanism Activating Nrf2-Keap1 Pathway in Regulation of Adaptive Response to Electrophiles. Free Radic Biol Med (2004) 36(10):1208–13. doi: 10.1016/j.freeradbiomed.2004.02.075 15110385

[48] Chipurupalli S Ganesan R Dhanabal SP Kumar MS Robinson N. Pharmacological STING Activation Is a Potential Alternative to Overcome Drug-Resistance in Melanoma. Front Oncol (2020) 10:758. doi: 10.3389/fonc.2020.00758 32477956 PMC7241280

[49] Corazao-Rozas P Guerreschi P Jendoubi M Andre F Jonneaux A Scalbert C. Mitochondrial Oxidative Stress is the Achille's Heel of Melanoma Cells Resistant to Braf-Mutant Inhibitor. Oncotarget (2013) 4(11):1986–98. doi: 10.18632/oncotarget.1420 PMC3875764 24161908

[50] Shimamoto K Hayashi H Taniai E Morita R Imaoka M Ishii Y. Antioxidant N-Acetyl-L-Cysteine (NAC) Supplementation Reduces Reactive Oxygen Species (ROS)-Mediated Hepatocellular Tumor Promotion of Indole-3-Carbinol (I3C) in Rats. J Toxicol Sci (2011) 36(6):775–86. doi: 10.2131/jts.36.775 22129741

[51] Sun Y Miao H Ma S Zhang L You C Tang F. FePt-Cys Nanoparticles Induce ROS-Dependent Cell Toxicity, and Enhance Chemo-Radiation Sensitivity of NSCLC Cells *In Vivo* and *In Vitro* . Cancer Lett (2018) 418:27–40. doi: 10.1016/j.canlet.2018.01.024 29331422

[52] Harvey KF Zhang X Thomas DM. The Hippo Pathway and Human Cancer. Nat Rev Cancer (2013) 13(4):246–57. doi: 10.1038/nrc3458 23467301

[53] Elosegui-Artola A Andreu I Beedle AEM Lezamiz A Uroz M Kosmalska AJ. Force Triggers YAP Nuclear Entry by Regulating Transport Across Nuclear Pores. Cell (2017) 171(6):1397–410.e14. doi: 10.1016/j.cell.2017.10.008 29107331

[54] Manning SA Kroeger B Harvey KF. The Regulation of Yorkie, YAP and TAZ: New Insights Into the Hippo Pathway. Development (2020) 147(8):dev179069. doi: 10.1242/dev.179069 32341025

[55] Zhao B Li L Lei Q Guan KL. The Hippo-YAP Pathway in Organ Size Control and Tumorigenesis: An Updated Version. Genes Dev (2010) 24(9):862–74. doi: 10.1101/gad.1909210 PMC2861185 20439427

[56] Ege N Dowbaj AM Jiang M Howell M Hooper S Foster C. Quantitative Analysis Reveals That Actin and Src-Family Kinases Regulate Nuclear YAP1 and its Export. Cell Syst (2018) 6(6):692–708.e13. doi: 10.1016/j.cels.2018.05.006 29909276 PMC6035388

[57] Johannessen CM Johnson LA Piccioni F Townes A Frederick DT Donahue MK. A Melanocyte Lineage Program Confers Resistance to MAP Kinase Pathway Inhibition. Nature (2013) 504(7478):138–42. doi: 10.1038/nature12688 PMC4098832 24185007

[58] Shalem O Sanjana NE Hartenian E Shi X Scott DA Mikkelson T. Genome-Scale CRISPR-Cas9 Knockout Screening in Human Cells. Science (2014) 343(6166):84–7. doi: 10.1126/science.1247005 PMC4089965 24336571

[59] Lin L Sabnis AJ Chan E Olivas V Cade L Pazarentzos E. The Hippo Effector YAP Promotes Resistance to RAF- and MEK-Targeted Cancer Therapies. Nat Genet (2015) 47(3):250–6. doi: 10.1038/ng.3218 PMC4930244 25665005

[60] Zhang X Yang L Szeto P Abali GK Zhang Y Kulkarni A. The Hippo Pathway Oncoprotein YAP Promotes Melanoma Cell Invasion and Spontaneous Metastasis. Oncogene (2020) 39(30):5267–81. doi: 10.1038/s41388-020-1362-9 32561850

[61] Sanchez-Hernandez I Baquero P Calleros L Chiloeches A. Dual Inhibition of (V600E)BRAF and the PI3K/AKT/mTOR Pathway Cooperates to Induce Apoptosis in Melanoma Cells Through a MEK-Independent Mechanism. Cancer Lett (2012) 314(2):244–55. doi: 10.1016/j.canlet.2011.09.037 22056813

[62] Shimizu T Tolcher AW Papadopoulos KP Beeram M Rasco DW Smith LS. The Clinical Effect of the Dual-Targeting Strategy Involving PI3K/AKT/mTOR and RAS/MEK/ERK Pathways in Patients With Advanced Cancer. Clin Cancer Res (2012) 18(8):2316–25. doi: 10.1158/1078-0432.CCR-11-2381 22261800

[63] Hartman ML Czyz M. Anti-Apoptotic Proteins on Guard of Melanoma Cell Survival. Cancer Lett (2013) 331(1):24–34. doi: 10.1016/j.canlet.2013.01.010 23340174

[64] Ribas A Gonzalez R Pavlick A Hamid O Gajewski TF Daud A. Combination of Vemurafenib and Cobimetinib in Patients With Advanced BRAFV600-Mutated Melanoma: A Phase 1b Study. Lancet Oncol (2014) 15(9):954–65. doi: 10.1016/s1470-2045(14)70301-8 25037139

[65] Keating GM. Cobimetinib Plus Vemurafenib: A Review in BRAF (V600) Mutation-Positive Unresectable or Metastatic Melanoma. Drugs (2016) 76(5):605–15. doi: 10.1007/s40265-016-0562-7 26984388

[66] Baudy AR Dogan T Flores-Mercado JE Hoeflich KP Su F van Bruggen N. FDG-PET Is a Good Biomarker of Both Early Response and Acquired Resistance in BRAFV600 Mutant Melanomas Treated With Vemurafenib and the MEK Inhibitor GDC-0973. EJNMMI Res (2012) 2(1):22. doi: 10.1186/2191-219X-2-22 22651703 PMC3405466

