# Supplementary material for: Dimethyl Fumarate Combined With Vemurafenib Enhances Anti-Melanoma Efficacy via Inhibiting the Hippo/YAP, NRF2-ARE, and AKT/mTOR/ERK Pathways in A375 Melanoma Cells
Source: Front Oncol. 2022 Jan 24;12:794216. doi: 10.3389/fonc.2022.794216 (PMC8820202; doi:10.3389/fonc.2022.794216)
Supplement: Supplementary file 1 [file DataSheet_1.docx]

Supplementary Material

**Supplementary Figures**

**Supplementary Figure 1**

**
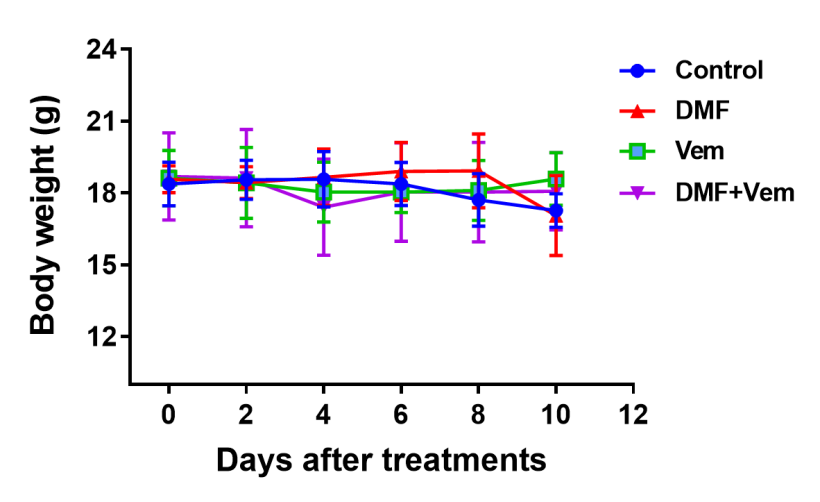
**

**Supplementary Figure 1| Body weight measurements over time.**

**Supplementary Figure 2**

**
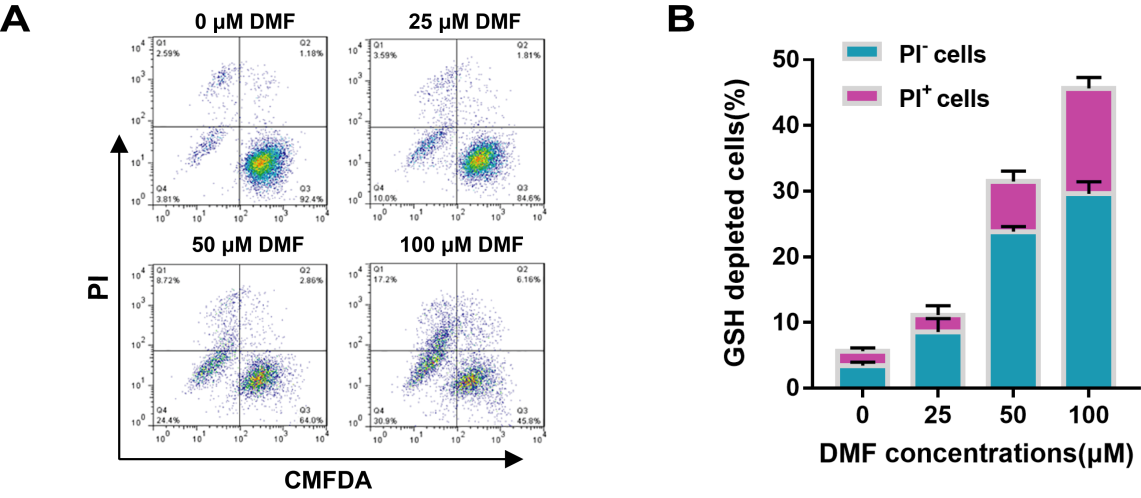
**

**Supplementary Figure 2| GSH levels are partially correlated with DMF-induced cell death in A375 melanoma cells. (A)** Representative flow cytometry profiles of A375 cells stained by CMFDA and PI. A375 cells were treated with the indicated doses of DMF for 24 h. **(B)** Quantitative and statistical analysis of the data from (A). Numerical data were from three independent experiments and shown as mean ± SD.

**Supplementary Figure 3**


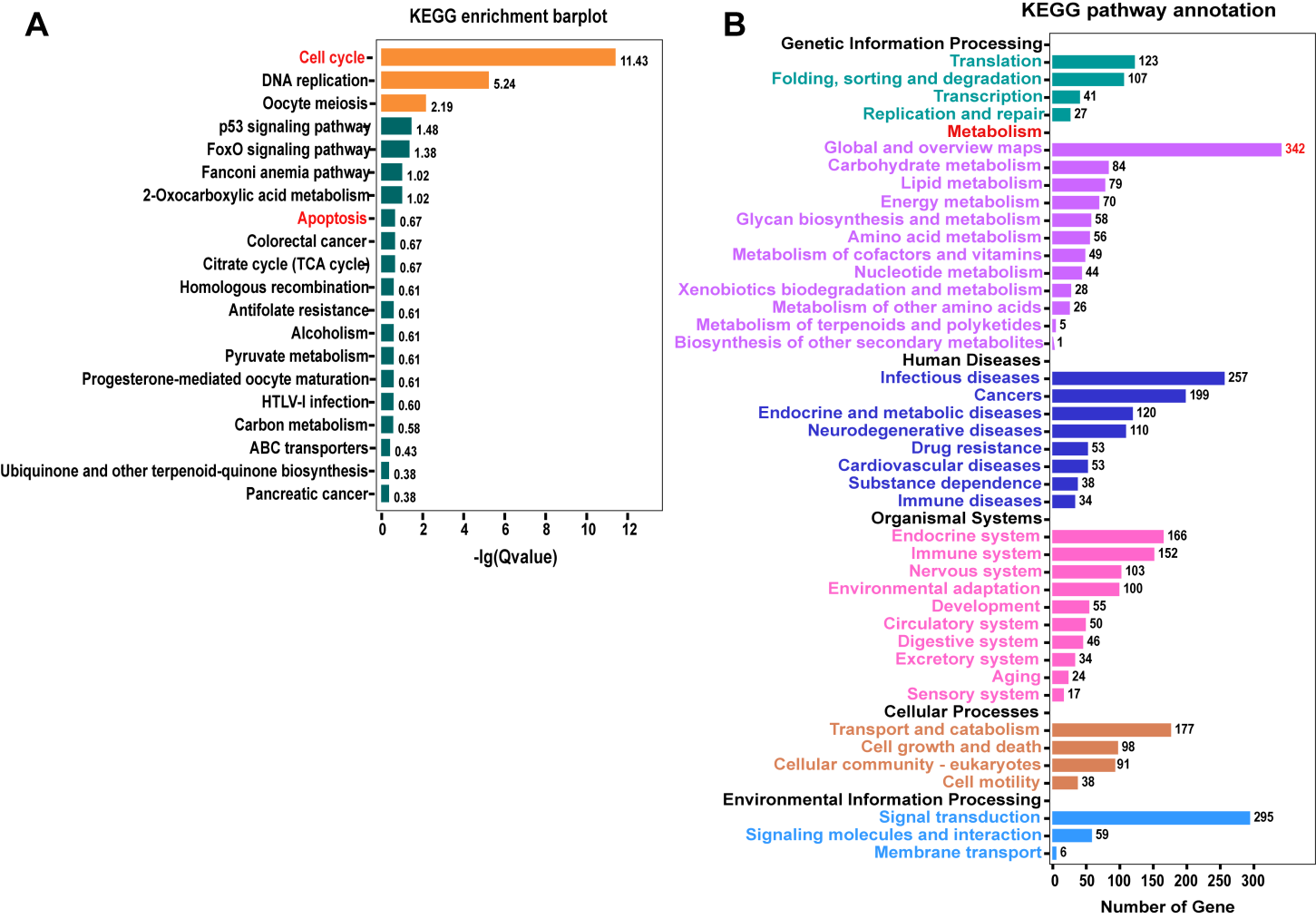


**Supplementary Figure 3| (A)** The top 20 enriched KEGG pathways identified through an analysis of DEGs that were shared among the monotherapy and combination therapy samples, *P*-value < 0.05. **(B)** KEGG pathway enrichment analysis of DEGs that were specifically upregulated in the context of DMF/Vem treatment.

**Supplementary Figure 4**

**
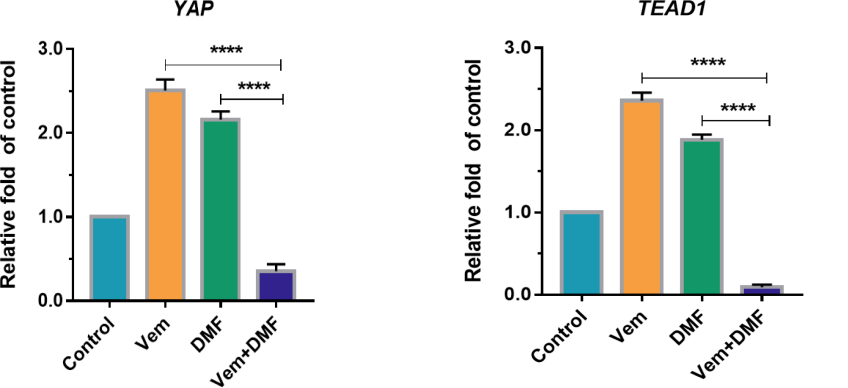
**

**Supplementary Figure 4| qPCR analysis results of Hippo signaling pathway-related genes.** A375 cells were treated with 50 μM of DMF and/or 2 μM of Vem for 24 h. qPCR was performed, with *18S* as a reference control; ****P* < 0.001, *****P* < 0.0001.

**Supplementary Figure 5**


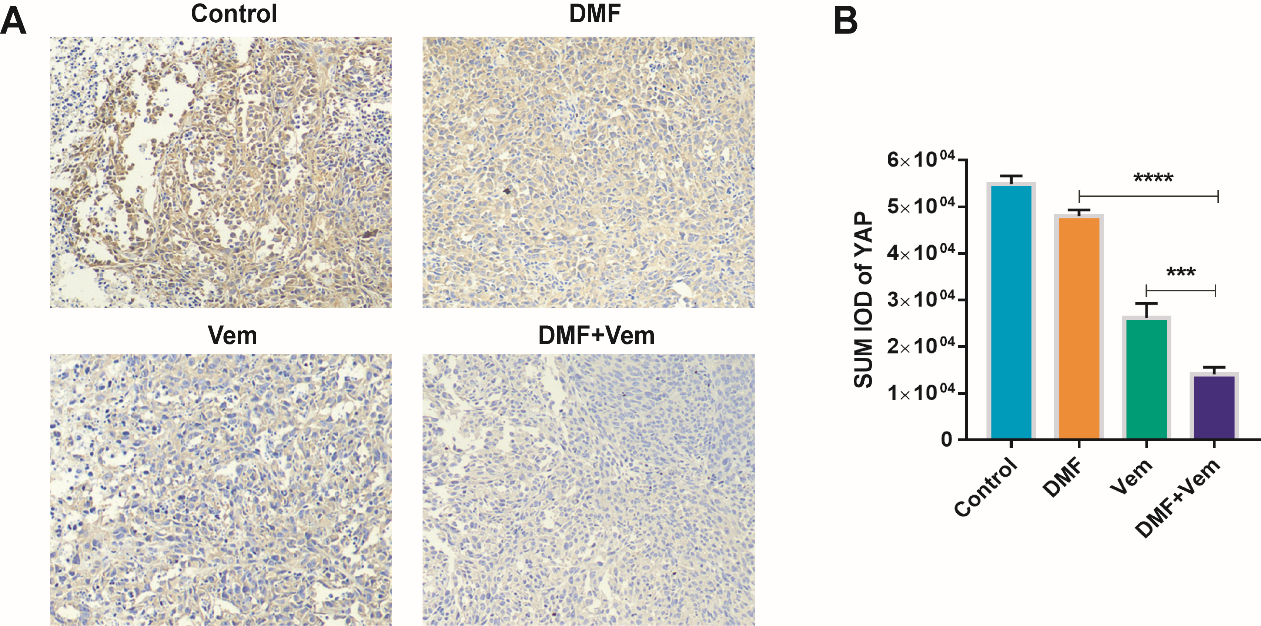


**Supplementary Figure 5|** **The immunohistochemical staining results of YAP. (A)** Representative images of YAP immunohistochemical staining in tumour tissue. All images were taken at 10× magnification. **(B)** The integrated optical density (IOD) of immunohistochemical results of YAP was calculated by Image Pro Plus 6.0. ****P <* 0.001, *****P <* 0.0001, using one-way ANOVA to compare the differences between the groups.

**Supplementary Table 1** The sequences of primers for qRT-PCR used in this study.

| Gene name | | Forward primer (5′ to 3′) | | Reverse primer (5′ to 3′) |
| --- | --- | --- | --- | --- |
| *YAP*  *TEAD1* | CTGCCCGACTCCTTCTTCAA  CCAACCATTCTTACAGTGACCCAT | | TGCAGAGAAGCTGGAGAGGAAT  TCAAACCTTGCATACTCCGTCTC | |
| *AKT1* | GACGGGCACATTAAGATCAC | | TGAGGATGAGCTCAAAAAGC | |
| *mTOR* | ATTTGATCAGGTGTGCCAGT | | GCTTAGGACATGGTTCATGG | |

*YAP*: Yes-associated protein; *TEAD1*: TEA domain transcription factor 1; *AKT1*: AKT serine/threonine kinase 1; *mTOR*: mechanistic target of rapamycin kinase.

**Supplementary Table 2** Antibodies used in the study.

| Name of antibody | Identifier | Source |
| --- | --- | --- |
| Phospho -AKT1/2/3 (Ser 473)  AKT1/2/3(H-136)  GAPDH(FL-335)  PARP-1(F-2)  Phospho-ERK1/2 (Thr202/Tyr204) | Cat#: sc-7985-R  Cat#: sc-8312  Cat#: sc-25778  Cat#: sc-8007  Cat#: 9101 | Santa Cruz  Santa Cruz  Santa Cruz  Santa Cruz  CST |
| ERK1/2 Antibody | Cat#: 9102 | CST |
| p70 S6 kinase α (H-9) Mouse mAb | Cat#: sc-8418 | Santa Cruz |
| Phospho-p70 S6 Kinase α (A-6) Mouse mAb | Cat#: sc-8416 | Santa Cruz |
| 4E-BP1 (53H11) Rabbit mAb | Cat#: 9644T | CST |
| Phospho-4E-BP1 (Thr37/46) Rabbit mAb | Cat#: 2855T | CST |
| YAP (D8H1X) XP^®^ Rabbit mAb | Cat#: 14074 | CST |
| TEF1/TEAD-1 Mouse monoclonal antibody | Cat#: ab133535 | Abcam |
| NRF2 Rabbit monoclonal antibody | Cat#: ab62352 | Abcam |
| HO-1/HMOX1 Rabbit polyclonal antibody | Cat#: 10701-1-AP | Proteintech |
| NQO1 Rabbit polyclonal antibody | Cat#: 11451-1-AP | Proteintech |
| Lamin B1 Rabbit polyclonal antibody | Cat#: 12987-1-AP | Proteintech |
| Alexa Fluor^TM^ 568 goat anti-rabbit IgG(H+L)  β-actin Mouse Monoclonal Antibody  HRP-labeled Goat Anti-Rabbit IgG(H+L)  HRP-labeled Goat Anti-Mouse IgG(H+L) | Cat#: A11036  Cat#: AF0003  Cat#:A0208  Cat#:A0216 | Invitrogen  Beyotime  Beyotime  Beyotime |

CST: Cell Signaling Technology.
